# Fee Bill

**Published:** 1873-02

**Authors:** 


The Fee-Bill.—Philadelphia stands alone among cities in the possession of a rigid fee-bill, carrying a certain weight of authority as having been adopted by the College of Physicians, and employed as a guide by many practitioners in making out their accounts. We have so recently alluded to the matter of compensation for medical services, that we need hardly go over the general ground again, but would urge once more the absurdity of assuming that the value of every man's professional attendance is the same, and to be charged for at the same rate. Skill, experience, reputation,—all these should be worth something pecuniarily, and the public would recognize the fact if the profession did. It is recognized by the army and navy authorities of every civilized country, in the increase of rank and pay accorded to medical officers in proportion to their length of service. Again, to reckon by visits only, without regard to the varying trouble and responsibility involved in different cases, or to the ability of the patients or their friends to pay, is manifestly absurd.

In point of fact, the fee-bill, if binding at all, is so only upon the Fellows of the College of Physicians. It is practically disregarded by many of them, and indeed, must be, unless those who attain success in business are willing to be overworked and underpaid. Lawyers have no such tariff; they charge what they can get,—that is, what the public consent to pay them for their services.

Let us suppose the fee-bill done away with entirely; who would be the worse off? Surely not the physician, for he knows well what his professional brethren consider the average value of their services, or he can readily find out; and he can tell what his investments in labor, time, money, and the acquisition of skill and experience, have been. As surely not the public; for they must, by an inexorable law of compensation, if they want underpaid doctors, have inefficient ones. We think the results of observation in other departments of skilled labor will show that, in the long run, good work must be well paid for, or the energy and ability it demands will flow into other channels.

It is earnestly to be hoped that this futile fee-bill may be abolished altogether at no distant day.—*Medical Times.*

